# Determining suitable surfactant concentration ranges to avoid protein unfolding in pharmaceutical formulations using UV analysis

**DOI:** 10.1016/j.heliyon.2023.e21712

**Published:** 2023-10-26

**Authors:** Laura J. Waters, Joseph Whiteley, William Small, Steve Mellor

**Affiliations:** a School of Applied Sciences, University of Huddersfield, Queensgate, Huddersfield, HD1 3DH, UK; b Croda Europe Ltd, Cowick Hall, Snaith, Goole, DN14 9AA, UK

**Keywords:** Biocompatibility, Critical micellar concentration, Pharmaceutical, Polysorbate, Protein, Spectroscopy, Tween, UV

## Abstract

Protein stability is fundamental to maintain pharmaceutical efficacy in the nascent field of biologics. One particular property that is essential for therapeutic effect is retention of the folded 3-dimensional conformation, i.e. once unfolding has occurred the biologic is often rendered inactive. In this work we propose a modified form of a recently published UV spectroscopic method that identifies protein unfolding. In this study we determine concentration limits to avoid protein unfolding of two model surfactants, namely polysorbate 20 and polysorbate 80, by correlating surfactant concentration with percentage ‘unfolded’ for three model proteins. For each scenario two distinct regions were observed, firstly surfactant concentrations at which no unfolding had occurred, followed by a second region whereby unfolding steadily increased with surfactant concentration. In general for the combinations analysed in this study, this second region began to appear around ten times below the critical micellar concentration of each surfactant, regardless of the protein or polysorbate chosen. It is therefore proposed that this adapted method could be used by researchers in the early stages of formulation development as a convenient and simple screening tool to confirm the ‘onset of unfolding’ concentration for protein-surfactant formulations, thus helping to optimise surfactant concentration selection in pharmaceutical formulations to maintain the benefits of surfactants yet avoid inadvertent unfolding.

## Graphical abstract

**Figure:**
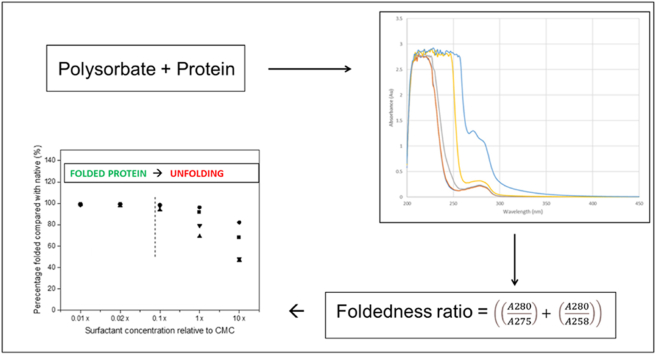

## 1 Introduction

Pharmaceutical therapies known as biologics are rapidly becoming the future of the pharmaceutical industry, as opposed to the previously utilised smaller and simpler drug molecules. Many biologics are protein-based including interleukins, interferons, hormones and polypeptides. Proteins co-exist in unfolded and native states and an equilibrium that favours the latter is often essential to maintain therapeutic efficacy and is referred to as conformational stability. The process of protein folding has been studied extensively, with respect to examining both the equilibrium and the kinetics of folding proteins [1]. Practical approaches to monitoring protein folding and assembly have incorporated a range of strategies, such as calorimetry [2], fluorescence emission spectroscopy, circular dichroism for structural changes [3,4], differential quenching [5] and small-angle neutron scattering [6]. An alternative aspect of protein stability often adopted by researchers is rather than consider the process of folding, instead monitor unfolding, for example using calorimetry [7] or fluorescence [[8], [9], [10]]. Unfortunately, each of these methods have associated experimental issues if they are to be applied in a quantitative manner. Firstly, circular dichroism is useful for qualitatively observing tertiary structures with near-UV CD bands and secondary structures with far-UV CD bands [11] yet quantitative estimates of stability involve lengthy extrapolation whereby reported discrepancies are observed when measured values are compared with calorimetric data [12]. Secondly, fluorescence-based methods either require the addition of a fluorophore [13] (which may affect the process being monitored) or a discernible difference in signal between the macroscopic states [14] which may not be the case in the presence of additional compounds, such as excipients. Thirdly, infrared spectroscopy is widely used to study protein structure, and has been applied quantitatively [15] yet, this method requires comparatively high protein concentrations, complex deconvolution of data through the analysis of second derivatives and again, potential interference from the addition of denaturing compounds (also an issue in calorimetric analysis). For these reasons, IR cannot be considered a convenient or simple method to quantify unfolding for more complex formulations. Finally, several other methods can also be used but are renowned for their complexity, such as neutron spectroscopy [16] or NMR [17]. This leaves measurement of unfolding from UV absorbance spectra as the only simple, expedient and easily accessible method suitable for quantitative analysis for proteins in the presence of additional compounds.

Pharmaceutical formulations contain not just the active ingredient but also a variety of excipients, particularly in small molecule pharmaceuticals, ranging from syloids [18] to polyols [19] to surfactants [20]. It has been claimed that there is on average half as many total excipients in parenteral biological formulations compared with oral small molecule medicines (4.5 vs. 8.8) [21], yet the impact of such excipients on the active ingredient can still be a cause for concern. This same study found that 29 % of biological formulations contained polysorbate 80 and that this surfactant, along with polysorbate 20 are the two most frequently used surfactants in biological formulations, available as Tween™ 20 and Tween™ 80 from Croda Europe Ltd. Polysorbate 20 and polysorbate 80 are used due to their non-ionic nature and low toxicity [21]. Their structure consists of a sorbitan molecule that has undergone esterification with polyoxyethylene (POE), and then further esterification of the POE chains with lauric acid for polysorbate 20 and oleic acid for polysorbate 80 [22]. Other researchers have reported higher rates of polysorbate incorporation in biopharmaceutical products [23], and thus the impact of their presence is of high importance to industry [24]. With a proven safety profile [25], polysorbates are incorporated in formulations to act as solubility enhancers, dispersion stabilisers and aggregation preventatives, often at concentrations in the range from 0.001 to 0.1 % w/v [26]. Surfactants are particularly useful as they can undergo a spontaneous process whereby monomer molecules rearrange to form micellar structures at a concentration specific to each surfactant, known as the critical micellar concentration (CMC). This process is thermodynamically driven and will be affected by the presence of other compounds in the solution, such as other excipients or the drug itself [27]. Furthermore, the properties of compounds will themselves be influenced by the inclusion of surfactants which can affect their ability to undergo processes such as permeation [28]. The interest in surfactants is a consequence of their wide variety of uses, such as their incorporation in pharmaceutical and cosmetic formulations, with recently developed ‘green’ surfactants becoming more widespread in agriculture, medicine, personal care and food products [29]. As previously mentioned, each surfactant has a specific CMC value which can be measured using a variety of techniques including surface tension, the addition of dye and more recently, isothermal titration calorimetry [30].

Proteins have been analysed extensively using UV–vis spectroscopy to measure the molar absorption coefficient [31], degradation [32], unfolding kinetics [33] as well as side groups within the protein structure [34]. Most interestingly, denaturation from unfolding has been studied using this form of analysis through monitoring absorbance changes as a consequence of pH changes [35], or with the addition of denaturants, such as urea [36]. The application of UV–vis analysis has also been used to study renaturation, i.e. refolding through reoxidation [37].

Of fundamental importance to this work is a study published in 2019 that utilised UV–vis analysis with a focus on the absorbance of three specific residues (tryptophan, tyrosine and phenylalanine at 280, 275 and 258 nm respectively) to calculate a ‘foldedness’ ratio [38]. In this pioneering study the unfolding and refolding of a protein was analysed using a sum of two ratios of absorbances ((A_280_/A_275_) + (A_280_/A_258_)) to consider the degree of unfolding observed. A logical progression of the aforementioned method has been applied in this study by extracting the concept and associated equation yet rather than monitoring unfolding, our study investigates a series of surfactant concentrations with three proteins to determine the concentration at which unfolding *begins* to occur. By using this published concept to identify the critical transformation point it is then possible to declare the concentration in a formulation before protein stability is compromised and the concentration window below which the presence of surfactant is not detrimental to therapeutic efficacy.

## 2 Materials and methods

### 2.1 Materials

Polyoxyethylene (20) sorbitan monolaurate (‘polysorbate 20’) and polyoxyethylene (20) sorbitan monooleate (‘polysorbate 80’) were kindly donated by Croda Europe Ltd. Four polysorbates were utilised: standard compendial grades, referred to as Tween™ 20 (BN:50702) and Tween™ 80 (BN:49659A), and high purity grades referred to as Super Refined™ Polysorbate 20 (BN:0001814116) and Super Refined™ Polysorbate 80 (BN:0001779440). The Super Refined versions are distinguishable from the standard grades with a series of benefits including a low peroxide value (2.0 meq O_2_/Kg max.), limited formaldehyde (10 ppm max.), low residual EO (1 ppm max.), low 1,4-dioxane (5 ppm max.), low residual Na and K (5 ppm max.), low moisture (0.2 % max.), decreased cellular irritation and microbial testing performed on each lot of material [22]. Three proteins were supplied by Sigma Aldrich, UK as lyophilised powders: immunoglobulin G (IgG) from human blood (>99 %), albumin from human serum (HSA) (>96 %) and β-lactoglobulin (β-Ig) from bovine milk (>90 %). These particular proteins were chosen to cover a range of molecular weights (and therefore sizes) from IgG (155 kDa) to HSA (65 kDa) to β-Ig (18 kDa). Potassium phosphate saline buffer of pH 7.4 was composed of 1.8 mM KH_2_PO_4_ (>99 %, Sigma Aldrich, UK), 8.2 mM K_2_HPO_4_ (>98 %, Sigma Aldrich, UK), 2.7 mM KCl (>99 %, Fisher Scientific, UK),140 mM NaCl (99.5 %, Acros Organics, UK) and ultra-pure water (18.2 MΩ cm).

### 2.2 Methods

Lyophilised protein powders were rehydrated by adding prepared buffer to the protein to achieve a concentration of 10 mg/mL, then stored at 5 °C for 72 h or until fully dissolved with the assumption aggregation was minimal at this stage. A series of polysorbate solutions were formulated based on multiples of the CMC recently reported for each individual surfactant [30]. These were: 10 x, 1 x, 0.1 x, 0.02 x and 0.01 x the CMC concentration for each of the four surfactants studied. To a 10 mL volumetric flask, 0.5 mL of protein solution was added to achieve a final protein concentration of 0.5 mg/mL, chosen to ensure the maximum absorbance value was always within measurable limits. A solution was produced for each surfactant at 10x the CMC (+5 % to account for the addition of 0.5 mL of protein solution). A serial dilution was then performed to achieve the remaining concentrations and added to each 10 mL volumetric flask. This was then repeated for the remaining two proteins analysed. UV spectroscopic analysis (Cary 60 UV–Vis, Agilent Technologies) with high precision quartz glass cells (Hellma Analytics, 10 mm light path) was used to analyse the absorbance of each sample at three specific wavelengths (258, 275 and 280 nm) using buffer (without polysorbate) as the blank and at 20 °C. Polysorbate in buffer was previously recorded to confirm no significant absorbance was visible in the analysed wavelength region (Supplementary Information Fig. S1) except for the highest concentration of Super Refined Tween 80 for phenylalanine whereby the polysorbate absorbance was subtracted from the protein absorbance prior to calculation. Each sample was measured in triplicate followed by a repeat analysis using fresh samples of both protein and surfactant solutions to determine the average absorbance values with associated error limits (n = 6). Averaged values were then analysed according to the equation proposed by Biter et al. [38] to facilitate calculation of the foldedness ratio for each sample which was then expressed as a percentage remaining folded in comparison with the value obtained with no surfactant present. From this it was possible to plot the relationship between surfactant concentration and the percentage of protein remaining folded to allow a visualisation of the concentration at which unfolding began to occur, thus identifying the acceptable polysorbate concentration range for formulation for each protein studied if unfolding is to be actively avoided.

## 3 Results and discussion

**Fig. 1:**
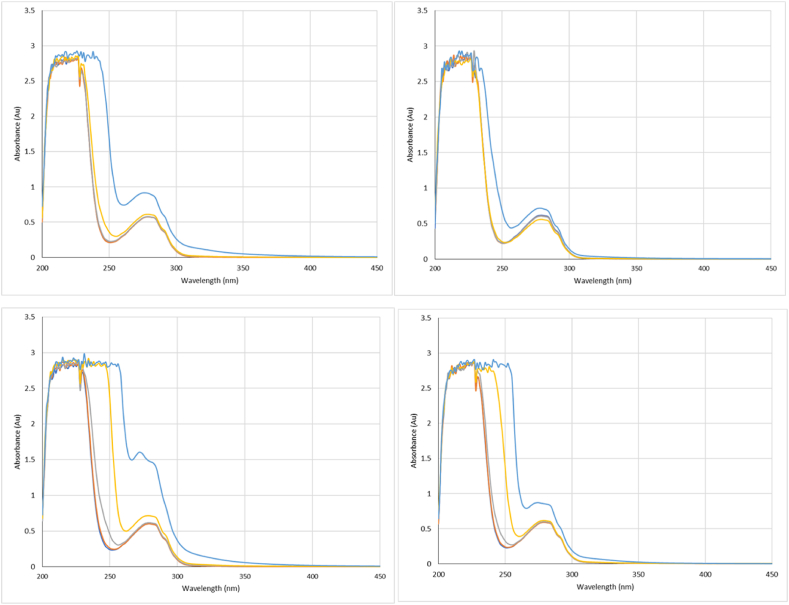
Absorbance spectra at the three specified wavelengths for IgG with Tween 20 (top left), Super Refined Polysorbate 20 (top right), Tween 80 (bottom left) and Super Refined Polysorbate 80 (bottom right). For each surfactant a series of concentrations were measured relative to each CMC: 0.01x (dark blue), 0.02x (orange), 0.1x (grey), 1x (yellow) and 10 x (light blue). (For interpretation of the references to colour in this figure legend, the reader is referred to the Web version of this article.)

**Fig. 2:**
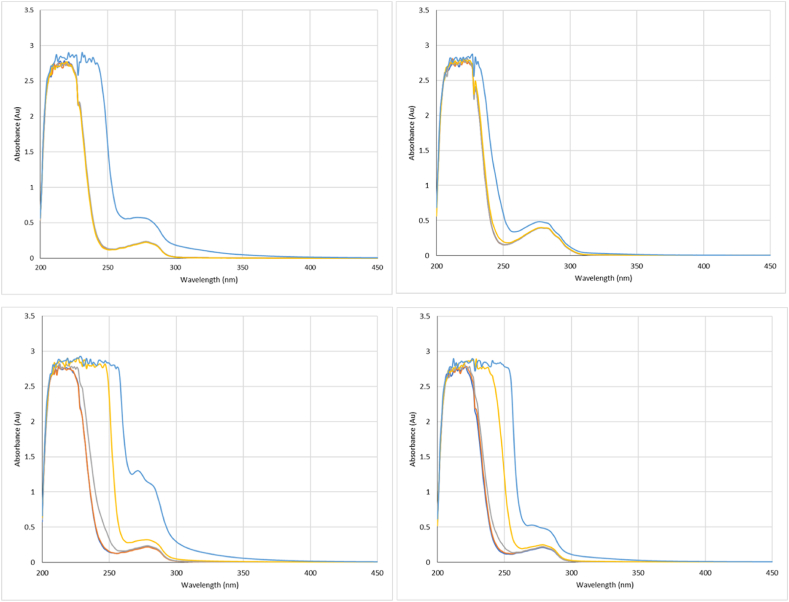
Absorbance spectra at the three specified wavelengths for HSA with Tween 20 (top left), Super Refined Polysorbate 20 (top right), Tween 80 (bottom left) and Super Refined Polysorbate 80 (bottom right). For each surfactant a series of concentrations were measured relative to each CMC: 0.01x (dark blue), 0.02x (orange), 0.1x (grey), 1x (yellow) and 10 x (light blue). (For interpretation of the references to colour in this figure legend, the reader is referred to the Web version of this article.)

**Fig. 3:**
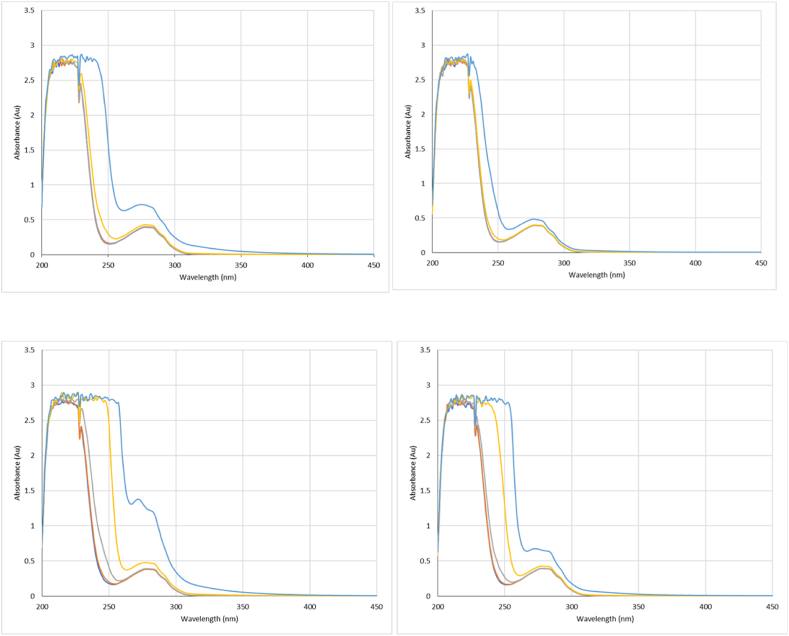
Absorbance spectra at the three specified wavelengths for β-Ig with Tween 20 (top left), Super Refined Polysorbate 20 (top right), Tween 80 (bottom left) and Super Refined Polysorbate 80 (bottom right). For each surfactant a series of concentrations were measured relative to each CMC: 0.01x (dark blue), 0.02x (orange), 0.1x (grey), 1x (yellow) and 10 x (light blue). (For interpretation of the references to colour in this figure legend, the reader is referred to the Web version of this article.)

Following acquisition of raw absorbance values at the three specified wavelengths for all four polysorbates (Fig. 1 (IgG), Fig. 2, Fig. 3 (β-Ig), the foldedness ratio was calculated for each surfactant concentration (Table 1 (IgG), Table 2 (HSA) and Table 3 (β-Ig)).

**Table 1:** Foldedness ratio calculations for IgG based on the absorbance values recorded in the presence of Tween 20 and Tween 80 (n ≥ 3, error = ±SD). CMC values extracted from literature^a^ = 2.4 mM,^b^ = 1.5 mM,^c^ = 2.5 mM,^d^ = 2.1 mM [30].

			Average absorbance (Au)			
Protein	Surfactant	Concentration relative to CMC	Tryptophan	Tyrosine	Phenylalanine	Folded ratio
IgG	Tween 20^a^	0.01x	0.5846	0.5652	0.2757	3.1552 (±0.0081)
		0.02x	0.5920	0.5718	0.2792	3.1560 (±0.0092)
		0.1x	0.5979	0.5783	0.2860	3.1244 (±0.0173)
		1x	0.6251	0.6089	0.3301	2.9203 (±0.0077)
		10x	0.9135	0.9244	0.768	2.1764 (±0.0124)
IgG	Super Refined Polysorbate 20^b^	0.01x	0.5967	0.5781	0.2819	3.1488 (±0.0075)
		0.02x	0.5890	0.5698	0.2790	3.1449 (±0.0101)
		0.1x	0.5891	0.5708	0.2796	3.1391 (±0.0111)
		1x	0.5760	0.5585	0.2829	3.0672 (±0.0085)
		10x	0.6976	0.6901	0.434	2.6169 (±0.0041)
IgG	Tween 80^c^	0.01x	0.5887	0.5691	0.2795	3.1404 (±0.0083)
		0.02x	0.5904	0.571	0.2832	3.1189 (±0.0037)
		0.1x	0.6045	0.5852	0.3087	2.9907 (±0.0064)
		1x	0.6892	0.6801	0.5820	2.1976 (±0.0275)
		10x	1.4483	1.5367	2.6497	1.4891 (±0.0041)
IgG	Super Refined Polysorbate 80^d^	0.01x	0.5935	0.5733	0.425	2.4285 (±0.0125)
		0.02x	0.6047	0.5846	0.2874	3.1382 (±0.0124)
		0.1x	0.5870	0.5677	0.2840	3.1010 (±0.0359)
		1x	0.6120	0.5962	0.4090	2.5230 (±0.0164)
		10x	0.8514	0.8674	0.5663	2.4846 (±0.0106)

**Table 2:** Foldedness ratio calculations for HSA based on the absorbance values recorded in the presence of Tween 20 and Tween 80 (n ≥ 3, error = ±SD). CMC values extracted from literature^a^ = 2.4 mM,^b^ = 1.5 mM,^c^ = 2.5 mM,^d^ = 2.1 mM [30].

			Average absorbance (Au)			
Protein	Surfactant	Concentration relative to CMC	Tryptophan	Tyrosine	Phenylalanine	Folded ratio
HSA	Tween 20^a^	0.01x	0.2258	0.2162	0.1357	2.7083 (±0.0209)
		0.02x	0.2256	0.2158	0.136	2.7032 (±0.0182)
		0.1x	0.2302	0.2200	0.1396	2.6956 (±0.0256)
		1x	0.2398	0.2311	0.1581	2.5546 (±0.1146)
		10x	0.5431	0.5623	0.6199	1.8420 (±0.0062)
HSA	Super Refined Polysorbate 20^b^	0.01x	0.2346	0.2270	0.1480	2.6189 (±0.0467)
		0.02x	0.2368	0.2281	0.1471	2.6481 (±0.0235)
		0.1x	0.2275	0.2178	0.1378	2.6948 (±0.0416)
		1x	0.2381	0.2288	0.1516	2.6117 (±0.0413)
		10x	0.3206	0.3219	0.2850	2.1207 (±0.0286)
HSA	Tween 80^c^	0.01x	0.2461	0.2355	0.1496	2.6904 (±0.0249)
		0.02x	0.2404	0.2296	0.1476	2.6756 (±0.0246)
		0.1x	0.2580	0.2478	0.1785	2.4860 (±0.0186)
		1x	0.3418	0.3407	0.4553	1.7542 (±0.0288)
		10x	1.1147	1.2129	2.6123	1.3457 (±0.0075)
HSA	Super Refined Polysorbate 80^d^	0.01x	0.2366	0.2256	0.1420	2.7148 (±0.0137)
		0.02x	0.2432	0.2321	0.1468	2.7042 (±0.0105)
		0.1x	0.2433	0.2324	0.1556	2.6108 (±0.0128)
		1x	0.2720	0.2643	0.2803	1.9997 (±0.0394)
		10x	0.4960	0.5192	0.4353	2.0949 (±0.0176)

**Table 3:** Foldedness ratio calculations for β-Ig based on the absorbance values recorded in the presence of Tween 20 and Tween 80 (n ≥ 3, error = ±SD). CMC values extracted from literature^a^ = 2.4 mM,^b^ = 1.5 mM,^c^ = 2.5 mM,^d^ = 2.1 mM [30].

			Average absorbance (Au)			
Protein	Surfactant	Concentration relative to CMC	Tryptophan	Tyrosine	Phenylalanine	Folded ratio
β-Ig	Tween 20^a^	0.01x	0.4213	0.4098	0.2085	3.0488 (±0.0175)
		0.02x	0.4380	0.4256	0.2154	3.0623 (±0.0045)
		0.1x	0.4443	0.4315	0.2210	3.0396 (±0.0080)
		1x	0.4227	0.4147	0.2404	2.7781 (±0.0166)
		10x	0.7144	0.7333	0.6777	2.0282 (±0.0124)
β-Ig	Super Refined Polysorbate 20^b^	0.01x	0.4048	0.3940	0.2010	3.0419 (±0.0211)
		0.02x	0.4106	0.3995	0.2033	3.0474 (±0.0089)
		0.1x	0.3934	0.3823	0.1947	3.0499 (±0.0196)
		1x	0.3933	0.3835	0.2031	2.9628 (±0.0101)
		10x	0.4814	0.4828	0.3388	2.4184 (±0.0111)
β-Ig	Tween 80^c^	0.01x	0.4017	0.3912	0.2021	3.0145 (±0.0098)
		0.02x	0.3946	0.3839	0.2009	2.9922 (±0.0069)
		0.1x	0.4017	0.3920	0.2253	2.8076 (±0.0036)
		1x	0.4819	0.4824	0.5082	1.9469 (±0.0218)
		10x	1.2323	1.3297	2.5875	1.4030 (±0.0071)
β-Ig	Super Refined Polysorbate 80^d^	0.01x	0.4171	0.4061	0.2092	3.0213 (±0.0084)
		0.02x	0.4068	0.3959	0.2040	3.0215 (±0.0064)
		0.1x	0.4085	0.3976	0.2145	2.9315 (±0.0110)
		1x	0.4387	0.4312	0.3424	2.2985 (±0.0057)
		10x	0.659	0.6831	0.4876	2.3164 (±0.0103)

These values were then converted to a percentage of protein remaining folded compared with the native sample. This was achieved by dividing the measured value by the value when no surfactant was present (IgG 3.1846, HSA 2.7445 and β-Ig 3.0270), expressed as a percentage. Error limits were calculated for all percentage values (±SD) and in all cases found to be <3.0 % with the exception of HSA with Tween 20 which has a SD of 6.2 %.

**Fig. 4:**
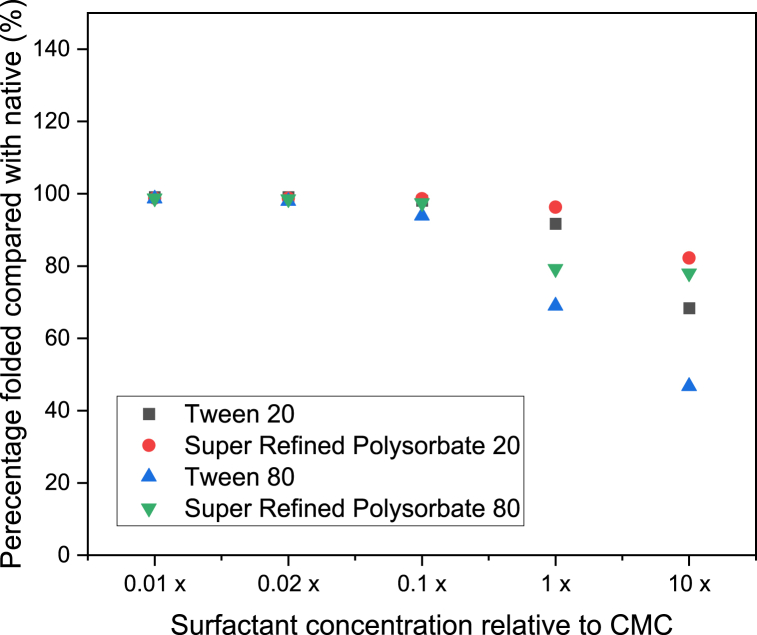
Percentage of IgG remaining folded (compared with native sample) with polysorbate concentration (relative to each CMC). (n ≥ 3, error = ±SD).

**Fig. 5:**
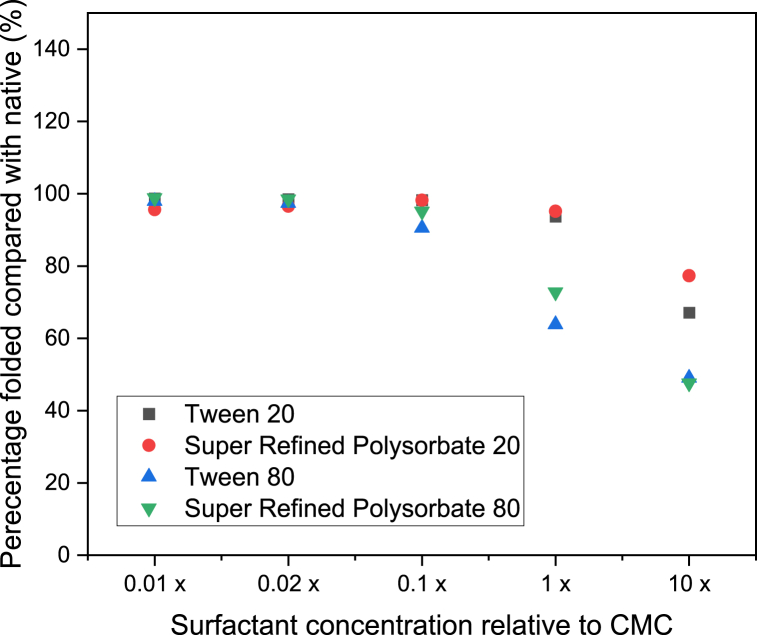
Percentage of HSA remaining folded (compared with native sample) with polysorbate concentration (relative to each CMC). (n ≥ 3, error = ±SD).

**Fig. 6:**
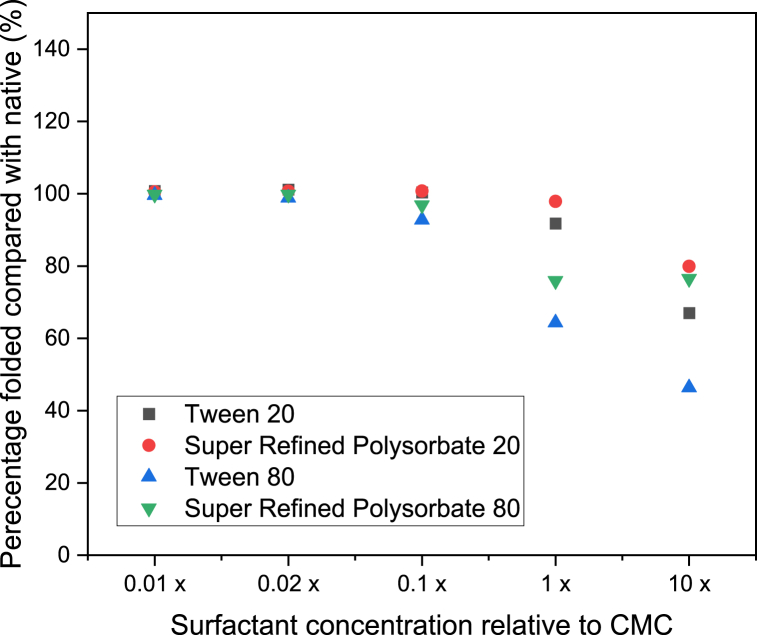
Percentage of β-Ig remaining folded (compared with native sample) with polysorbate concentration (relative to each CMC). (n ≥ 3, error = ±SD).

This facilitated a plot for each protein whereby the percentage remaining folded was plotted with surfactant concentration for IgG (Fig. 4), HSA (Fig. 5) and β-Ig (Fig. 6).

A decrease in the folded ratio indicates unfolding and occurs when there is a decrease in the tryptophan absorbance, or an increase in the tyrosine and/or phenylalanine absorbance, relative to that observed for the native state. Previous work has found that the foldedness ratio is inversely correlated with residual activity and therefore calculation of the absolute difference between the native and non-native forms can be an indication of protein structure [38]. From Fig. 4 for IgG it can be seen that at one hundred times and fifty times below the CMC for all four polysorbates, the percentage remaining folded (compared with the native state) remains approximately 100 %. However, at ten times below each surfactant CMC small differences in the percentage remaining folded begin to appear. This effect is exacerbated as the surfactant concentration continues to increase past the CMC and up to ten times the CMC. From reviewing the raw absorbance data at the three specific wavelengths required for calculation it can be seen that the absorbance at all three wavelengths increases. However, it is apparent that the reduction in percentage folded is a consequence of a more dramatic increase in the absorbance values at 258 nm which then causes an overall reduction in the total value calculated. Thus, it can be concluded that the unfolding process facilitates an increase in the exposure of phenylalanine groups. For HSA (Fig. 5) and β-Ig (Fig. 6) a similar profile was observed in that unfolding began to occur at ten times below the CMC of each surfactant in all cases although the effect was slightly more pronounced for these two proteins, compared with IgG. Amongst the four polysorbates the same profile was observed for two of the proteins (IgG and β-Ig) in that both standard grades of Tween resulted in more unfolding than both Super Refined forms. This difference implies the Super Refined forms provide more resilience to unfolding compared with the standard grade versions. Differences observed between Tween 20 and Tween 80 can be explained by considering the surfactants themselves. These non-ionic surfactants have ethoxylate groups that interact with hydrophobic moieties and hydrophobic cavities of proteins, this interaction can cause exposure of hydrophilic groups present in both molecules. This results in an increase in the hydrophilicity of the non-ionic surfactant–protein complex, with the advantageous benefit of reducing the aggregation of proteins [39]. Researchers have proposed that the slightly smaller volume calculated for Tween 20 compared with Tween 80 accounts for the small differences observed in their work [40] and fits with the results presented in this study. It can therefore be concluded that Tween 80 is larger and also more hydrophobic than Tween 20 (as reflected in their HLB values of 15.0 and 16.7 respectively [41]), hence causes more disruption to the folded protein structure and subsequently causes comparatively more unfolding to occur. Differences between the standard grade and Super Refined Polysorbates 20 and 80 are likely to be a result of the superior level of purity in the latter form.

Although it is of general scientific interest to observe how polysorbate concentration influences unfolding, the main objective of the study was to validate the applicability of the method to determine its suitability as a screening tool to investigate the range of surfactant concentrations that can be employed in pharmaceutical formulations. From the data presented regarding the formulations analysed in this study, it can be concluded that surfactant concentrations up to a maximum of ten times lower than the CMC can be added during formulation to provide drug solubilisation, avoid aggregation and yet also preserve protein conformation. For combinations of surfactant and protein analysed in this study the concentration below which unfolding occurred is ten times less than the CMC values recently reported [30], i.e. 0.03, 0.02, 0.03 and 0.03 % w/v for Tween 20, Super Refined Polysorbate 20, Tween 80 and Super Refined Polysorbate 80 respectively. This finding highlights that careful thought should be put into choosing a suitable surfactant concentration during the formulation development process to provide the advantages of addition yet avoid unintentional protein unfolding occurring. It is proposed that this form of screening analysis is a fast, simple, inexpensive, and therefore ideal way to help researchers create the most optimal formulations possible where surfactants and proteins are to be co-formulated. Of additional importance is the ability of this method to quantify unfolding which is not possible using alternative methods. Experiments were undertaken (data not shown) to analyse profiles of the proteins with and without surfactants at concentrations equal to those used in this study. In all three forms of analysis attempted (differential scanning calorimetry, fluorescence spectroscopy and Fourier transform infrared spectroscopy) the presence of the surfactant masked the unfolding phenomenon making it impossible to quantify the process. It is proposed that a similar result would also be observed if other techniques were attempted, such as circular dichroism or NMR as the presence of the surfactant interferes with the profile recorded. It is the unique sensitivity to absorbance separation that UV analysis provides that makes quantitative analysis the only suitable method in these situations.

## 4 Conclusion

In summary, a modified form of a recently published UV spectroscopic technique to determine protein unfolding was developed and validated to determine concentration ranges to avoid protein unfolding by correlating surfactant concentration with percentage ‘unfolded’ for three model proteins. For each scenario unfolding began to appear around ten times below the critical micellar concentration of each surfactant, regardless of the protein or polysorbate chosen. It is therefore proposed that this method could be used as a screening tool to confirm the ‘onset of unfolding’ concentration for other protein-surfactant formulations to maintain the benefits of surfactants yet avoid inadvertent unfolding. Furthermore, calculation using UV data is the sole form of analysis that can facilitate quantitative unfolding values for complex formulations such as this.

## Credit author statement

Laura Waters: Conceptualisation, Methodology, Validation, Resources, Writing – original draft, Writing – review & editing, Visualisation, Supervision, Project administration, Funding acquisition. Joseph Whiteley: Methodology, Validation, Investigation, Writing – review & editing. William Small: Methodology, Writing – review & editing, Supervision, Funding acquisition. Steve Mellor: Writing – review & editing, Funding acquisition.

## Data availability statement

Data will be made available on request.

## Ethics declaration

Review and/or approval by an ethics committee was not needed for this study because it did not meet such requirements based on the research involved.

## Declaration of competing interest

The authors declare that they have no known competing financial interests or personal relationships that could have appeared to influence the work reported in this paper.
