# Supplementary material for: Determining suitable surfactant concentration ranges to avoid protein unfolding in pharmaceutical formulations using UV analysis
Source: Heliyon. 2023 Oct 26;9(11):e21712. doi: 10.1016/j.heliyon.2023.e21712 (PMC10632529; doi:10.1016/j.heliyon.2023.e21712)
Supplement: Multimedia component 1 [file mmc1.docx]

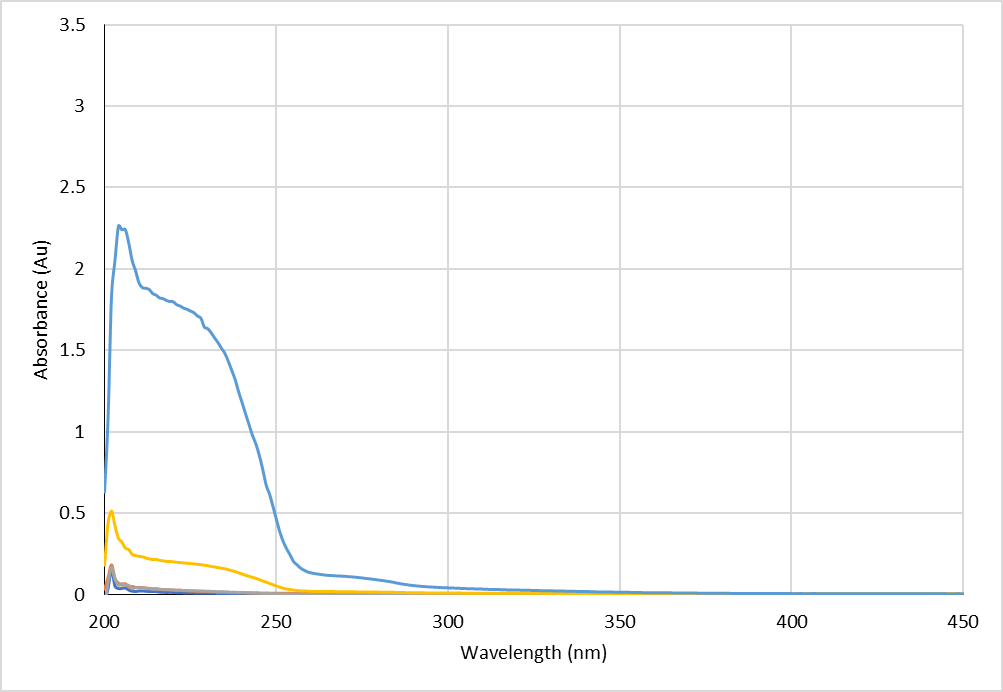

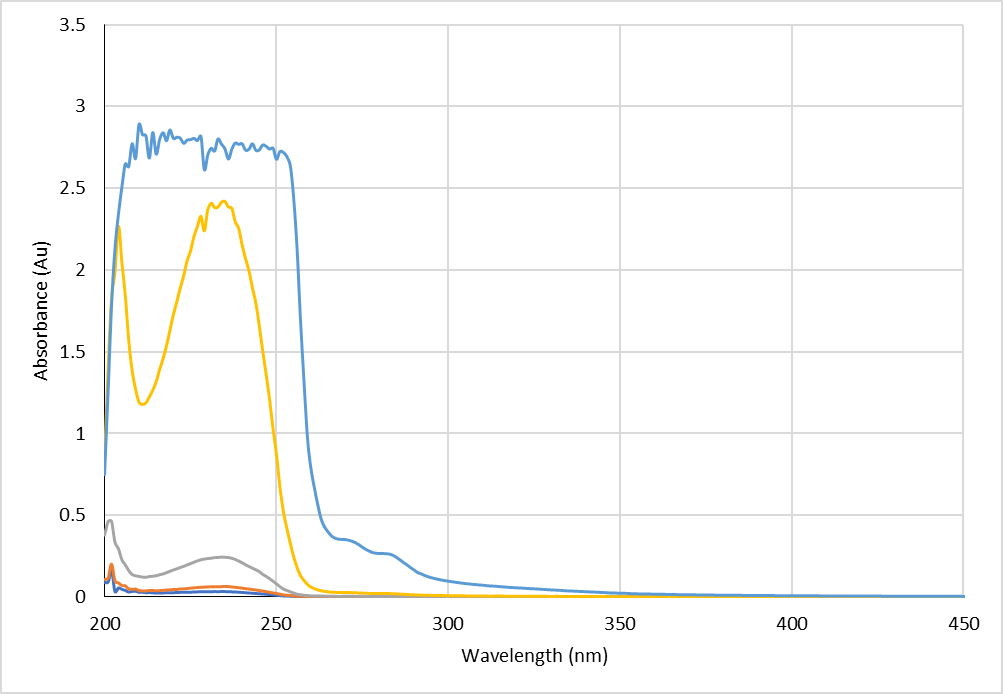

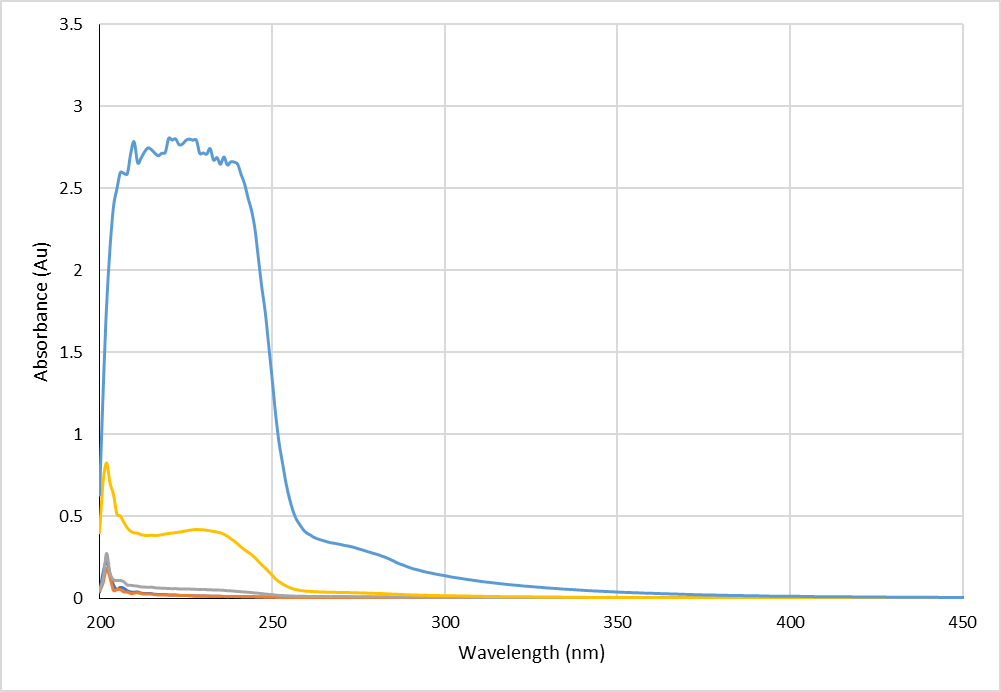


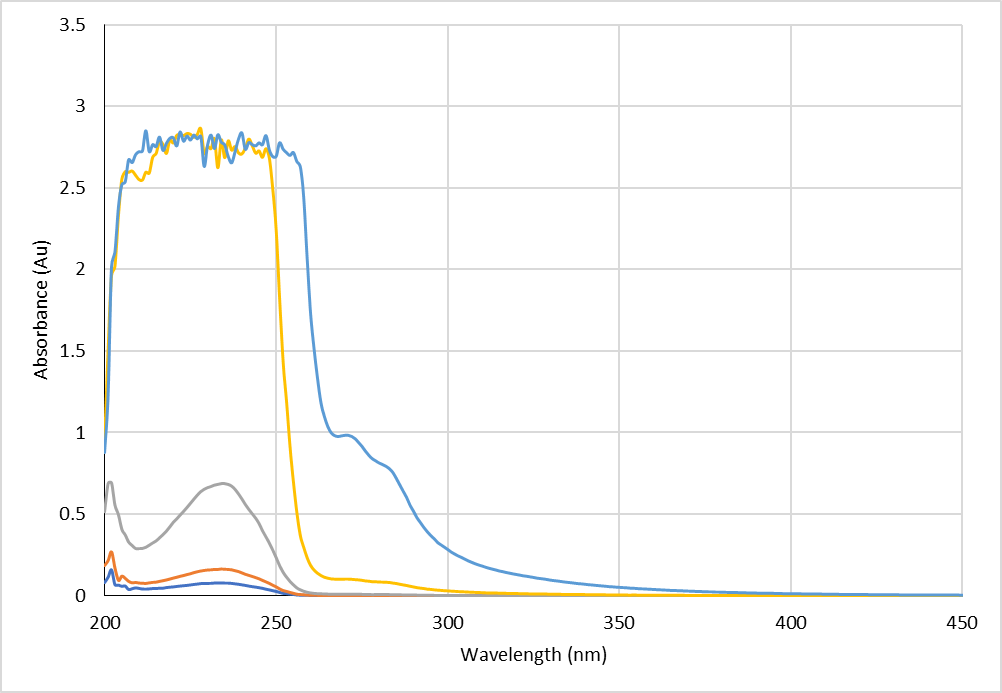


Figure S1: Absorbance spectra for Tween 20 (top left), Super Refined Polysorbate 20 (top right), Tween 80 (bottom left) and Super Refined Polysorbate 80 (bottom right). For each surfactant a series of concentrations were measured relative to each CMC: 0.01x (dark blue), 0.02x (orange), 0.1x (grey), 1x (yellow) and 10 x (light blue).
